# SMILES2Docking: An Open‐Source Desktop Workflow for Ligand Ionization, Stereochemistry‐Aware Preparation and Semi‐Empirical 3D Refinement

**DOI:** 10.1002/minf.70053

**Published:** 2026-09-07

**Authors:** Daniel Andrés Grajales Ruiz, Bruna Flôres Negrisoli, Isabel Cristina Conceição Periquito, Nailton Monteiro do Nascimento‐Júnior, Adriano Marques Gonçalves

**Affiliations:** ^1^ Laboratory of Medicinal Chemistry Organic Synthesis and Molecular Modeling (LaQMedSOMM) Department of Biochemistry and Organic Chemistry Institute of Chemistry São Paulo State University (UNESP) Araraquara São Paulo Brazil; ^2^ College of Veterinary Medicine and Animal Science University of São Paulo (USP) São Paulo São Paulo Brazil; ^3^ Department of Health and Biological Science University of Araraquara (UNIARA) Araraquara São Paulo Brazil

**Keywords:** ligand preparation, molecular docking, open‐source software, semi‐empirical methods, SMILES

## Abstract

SMILES2Docking converts spreadsheet‐based SMILES libraries into docking‐ready 3D ligands through a single, configurable pipeline. Salt and coformer removal, a three‐mode stereocenter policy, prediction of the dominant protonation state with a graph neural network pKa model (MolGpKa) refined by iterative titration, distance‐geometry embedding with RDKit, a molecular‐mechanics optimization cascade, and an optional semi‐empirical refinement under an implicit solvation model are combined in one tool, with records processed either sequentially or distributed across CPU cores. Users select among permissive, strict, and enumerative stereocenter modes, so the same tool serves broad exploratory screening and precision docking. A per‐compound JSON audit report records the ionization and stereocenter decisions taken for every structure. The tool is distributed as a Python package, a Windows executable with bundled MOPAC, a Linux portable bundle, and a macOS bundle, under GPL‐2.0‐or‐later, and is freely available to non‐commercial users at https://github.com/amgoncalvesusp/Smiles2Docking (DOI: 10.5281/zenodo.20617898).

## Graphical abstract

SMILES2Docking is an open‐source desktop workflow that converts spreadsheet SMILES libraries into docking‐ready 3D ligands. A single auditable pipeline performs structural curation, stereochemistry control, MolGpKa pH‐dependent ionization, molecular mechanics geometry, and optional semi‐empirical MOPAC refinement, and exports MOL2, SDF, or PDBQT files.

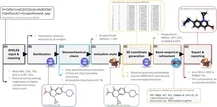

1

Virtual screening campaigns routinely interrogate libraries of 10^5^–10^9^ compounds described as SMILES strings [1], drawn from public repositories such as ChEMBL [2], ZINC [3], Enamine REAL, and PubChem [4]. Molecular docking, which has supported structure‐based drug discovery since the 1970s, now extends well beyond the analysis of individual ligand‐receptor complexes to include large‐scale virtual screening, drug repositioning, polypharmacology, and in silico toxicity profiling [5]. A recent bibliometric study reported an annual growth rate of 29.7% in docking‐related publications over the last decade, with a marked concentration in emerging research economies [6]. The integration of machine‐learning scoring functions and hybrid computational strategies has further increased throughput; within this workflow, the quality of the input ligand structures is one determinant of the final result, alongside the scoring function, the receptor model, and the definition of the binding site [7, 8, 9].

Several open‐source pipelines already automate parts of this process. VirtualFlow [10], DockStream [11], EasyDock [12], and DockM8 [13], among others, couple ligand preparation to one or more docking engines and target large‐scale virtual screening, and they rely on established components for the individual preparation subtasks (Table 1); the principal methodological difficulty lies in selecting and validating an appropriate method for each subtask rather than in assembling the pipeline itself. In most of these tools, the preparation stage is tied to a particular docking backend and is configured through scripts or command‐line options, which suit automated high‐throughput campaigns but offer limited visibility into the per‐compound decisions taken during preparation. SMILES2Docking occupies a complementary position. Rather than performing docking, it concentrates on the preparation stage as an auditable, engine‐independent step in which the stereochemistry policy and the ionization parameters are surfaced as explicit user choices and recorded for every structure, and it exposes this functionality through a desktop graphical interface as well as a command line. A graphical interface is not unique to this tool, as DockM8 also provides one; the distinguishing emphasis here is the decoupling of an auditable preparation stage from the docking engine, together with the per‐compound record of ionization and stereochemistry decisions. This focus on transparency and accessibility is intended to serve both research applications, in which reproducibility and a correct, physically ranked ionization state affect the downstream docking result, and educational or resource‐limited settings, in which accessibility and auditability are valued, while the prepared ligands remain compatible with any of the docking pipelines mentioned above.

**TABLE 1:** Representative open‐source tools for each ligand‐preparation subtask, and the methods adopted in SMILES2Docking.

Preparation subtask	Representative open‐source tools	Adopted in SMILES2Docking
Stereoisomer enumeration	RDKit EnumerateStereoisomers	RDKit
Protonation‐state assignment	Open Babel, Dimorphite‐DL, MolGpKa, Uni‐pKa	MolGpKa (default), Dimorphite‐DL, Open Babel
Tautomer generation and ranking	RDKit TautomerEnumerator, sPhysNet‐Taut	RDKit enumeration + sPhysNet‐Taut (optional)
3D coordinate generation	RDKit ETKDG, Open Babel, CORINA (commercial)	RDKit ETKDGv3
Geometry optimization	MMFF/UFF force fields, MOPAC PM6/PM7	MMFF94 to MMFF94s to UFF cascade, optional MOPAC PM7

Converting a one‐dimensional SMILES string into a docking‐ready three‐dimensional ligand requires several transformations that are individually well established, namely, the removal of salts and of disconnected components such as counter‐ions and co‐formers (species that accompany the active molecule in a multicomponent crystal), the handling of unspecified stereocentres, the assignment of the dominant ionization state at the biologically relevant pH, the generation of initial three‐dimensional coordinates, and geometry refinement to a level compatible with scoring functions [14]. The difficulty resides less in executing each step than in selecting, for each of them, a reliable method and in combining the outcomes into a reproducible and auditable procedure. Two of these steps are particularly error‐prone. When undefined chirality is ignored, the embedding engine assigns one arbitrary configuration; the resulting pose is physically valid for that stereoisomer, yet it may represent a stereochemical form other than the intended one, which weakens the interpretation of the docking result [15]. Neglecting the pH dependence of ionization likewise degrades the conformational search and scoring accuracy [9]. Mature open‐source toolkits address individual steps: RDKit [16] for coordinate generation and stereochemistry perception, Dimorphite‐DL [17] for pH‐dependent ionization, and Open Babel [18] as an alternative for the same task; yet assembling a complete and auditable preparation pipeline still requires user‐written orchestration across heterogeneous interfaces. Commercial alternatives such as LigPrep, OMEGA [19], and CORINA [20] integrate several of these steps but impose licensing costs that hinder reproducibility in academic and resource‐limited settings, particularly in developing countries. In practice, both the stereochemistry policy and the ionization parameters are often buried inside scripting code rather than exposed as auditable, user‐level decisions, and the gap widens when a semi‐empirical geometry refinement is desired but no convenient integration exists. The requirement for a precomputed three‐dimensional ligand structure is specific to conventional docking; recent co‐folding methods such as AlphaFold3 [21] and Boltz‐2 [22] predict the bound complex directly from the sequence and chemical identity and do not require prior three‐dimensional ligand generation, a context in which the present workflow is not intended to operate.

This Application Note describes SMILES2Docking, an open‐source workflow distributed under the GPL‐2.0‐or‐later license that converts SMILES‐based compound libraries into docking‐ready ligands through a single, configurable pipeline. The workflow exposes a flexible stereochemistry policy as an explicit user choice: molecules with undefined tetrahedral centers and alkene stereochemistry may be processed without restriction for broad exploratory screening of large public databases, restricted to fully defined stereocentres only for rigorous precision docking, or expanded by enumerating all possible stereoisomers up to a configurable cap. The dominant ionization state is predicted at a user‐defined pH by MolGpKa, a graph‐convolutional pKa model refined by iterative titration, with rule‐based enumeration through Dimorphite‐DL and Open Babel available as alternative backends, and an optional dominant‐tautomer selection may precede ionization, and three‐dimensional coordinates are generated via RDKit's distance‐geometry algorithm followed by a force‐field optimization cascade, with optional refinement by MOPAC PM7, or another semi‐empirical method distributed with MOPAC, under an implicit solvation model [23]. A machine‐readable audit report accompanies every run, recording per‐structure stereochemistry decisions, ionization states, and any charge mismatches introduced during the refinement. The application is distributed as a Python source tree, a Windows executable with a bundled MOPAC binary for zero‐configuration use, and a Linux and macOS portable bundle, which may be used with a pre‐installed MOPAC. SMILES2Docking is freely available at https://github.com/amgoncalvesusp/Smiles2Docking (DOI: 10.5281/zenodo.21379746).

The implementation runs in Python 3.11, either as a PySide6 desktop application or from the command line, with both interfaces sharing the same configuration. As shown in Figure 1A, input is read from SMI, CSV, TSV, XLS, or XLSX spreadsheets via pandas; column names are matched case‐insensitively, so input files need no reformatting. Each record carries its original row index through the entire pipeline, preserving a direct link between the spreadsheet and the exported structure.

**FIGURE 1:**
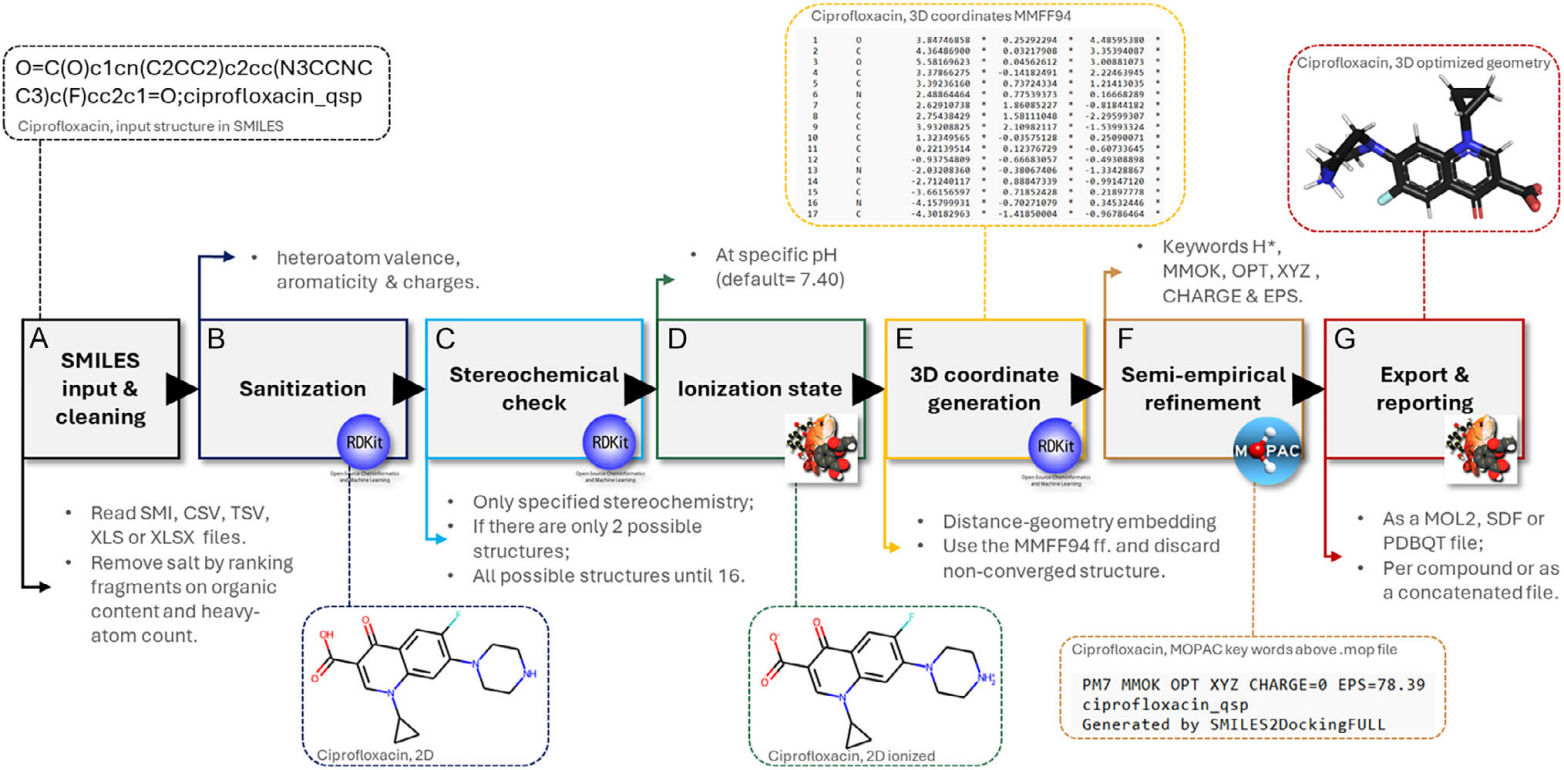
Step‐by‐step methodology of SMILES2Docking for three‐dimensional structure generation and semi‐empirical refinement. Atoms of hydrogen are shown in white, carbon in black and oxygen in red for three‐dimensional structures. ff, force field.

SMILES strings encoding multiple disconnected fragments (typical for salts and co‐crystallized counter‐ions) are handled by ranking fragments on organic content and heavy‐atom count, keeping the top‐scoring one (see Figure 1A). When two fragments score identically, and conservative mode is active, the run halts with an explicit error rather than guessing. In a large batch over a public library, a silent wrong choice would propagate unnoticed into every downstream step, so the failure is deliberate. Figure 1B summarizes the sanitization step for SMILES strings, where the verification of correct aromaticity assignments for all atoms and the valence/charge on N and O atoms is applied.

The stereocenter policy is then applied, with three modes available, as shown in Figure 1C. Permissive mode passes all molecules regardless of whether their tetrahedral and alkene stereocenters are assigned, the appropriate default when working from databases like ChEMBL or PubChem, which contain many structures with incomplete stereo annotation. Strict mode rejects any compound with one or more undefined tetrahedral and/or alkene stereocenters, limiting the output to geometrically unambiguous structures. The enumerative mode generates all possible diastereomers up to a configurable cap (default = 16) and tags each variant with a unique identifier derived from the parent code, so all downstream files remain individually traceable.

As shown in Figure 1D, the dominant ionization state at a user‐specified pH (default 7.4) is assigned by MolGpKa [24], a graph‐convolutional network that predicts the microscopic pKa of each ionizable atom. Independent per‐atom prediction on the neutral molecule does not capture the electrostatic coupling between neighboring ionizable centers; the dominant microstate is therefore obtained by iterative titration, in which the most favorable site is protonated or deprotonated, the pKa values are re‐predicted on the resulting charged species, and the procedure is repeated until no further change is favored, so that a single, physically ranked protonation state is returned. A per‐atom chemical prior additionally corrects the systematic under‐prediction of MolGpKa for small, unactivated aliphatic amines, a chemotype under‐represented in its training data, restoring their expected protonation at physiological pH without affecting amines that bear an electron‐withdrawing group or that are coupled to an already charged center. For piperazine at pH 7.4, the procedure yields the mono‐cation, consistent with macroscopic pKa values of approximately 9.8 and 5.6, whereas the single‐pass hydrogen addition of the Open Babel ‐p mode [18] returns the di‐cation and the rule‐based enumeration of Dimorphite‐DL [17] returns the full set of plausible states without ranking a dominant one (Table 2). Rule‐based approaches, whether the ‐p mode of Open Babel or the SMARTS rules of Dimorphite‐DL, share a conceptual limitation since they cannot represent long‐range electronic effects or the coupling between multiple ionizable centers, and this constraint affects acidic as well as basic groups. Dimorphite‐DL is retained as an explicit enumeration backend, which returns every plausible state within a configurable pH window and exports one structure per state, and Open Babel is retained for compatibility with legacy pipelines. The selection of a suitable open‐source protonation method is itself not straightforward, and a recent comparison within the EasyDock framework [25] provides further context [26]. When the resulting SMILES cannot be re‐parsed by RDKit, an SDF intermediate is used instead. The formal charge of the ionized species is recorded and carried into the post‐refinement charge audit (see Figure 1F).

**TABLE 2:** Protonation state assigned at pH 7.4 by each backend. MolGpKa returns a single, physically ranked dominant microstate; Open Babel returns one state from a single‐pass heuristic; Dimorphite‐DL enumerates all plausible states without ranking a dominant one. For piperazine, only MolGpKa returns the expected mono‐cation. The additional column reports the prediction of the original MolGpKa implementation, which applies Henderson‐Hasselbalch independently at each site; the two implementations differ only for piperazine, protonated at both nitrogens (+2) by the original model and returned as the mono‐cation (+1) by the iterative titration used here. A comparison based on the total formal charge is only partially informative, since different methods may protonate different centers and still yield the same overall charge, so agreement in net charge does not guarantee an identical microstate.

Molecule	MolGpKa (original)	MolGpKa (this work; dominant)	Open Babel ‐p	Dimorphite‐DL (states; charges)
Piperazine	+2	+1	+2	3 states; {0, +1, +2}
Acetic acid	−1	−1	−1	1 state; {−1}
Benzoic acid	−1	−1	−1	1 state; {−1}
Imidazole	0	0	0	4 states; {−1, 0, +1}
Morpholine	+1	+1	+1	2 states; {0, +1}
Histamine	+1	+1	+1	4 states; {−1, 0, +1, +2}

RDKit then generates 3D coordinates from a distance‐geometry algorithm [27]. The workflow summarized in Figure 1E starts with reproducible embedding at a fixed random seed; if that fails, it tries a random seed variant, then a small‐ring‐adapted variant for strained or macrocyclic systems, with multiple attempts at each stage before moving on. The embedded geometry is minimized through successive cycles of the molecular mechanics force field MMFF94 [28, 29] until convergence. If no force field converges, the record is rejected, and the user is informed in the final log.

Semi‐empirical refinement is disabled by default. When PM7 refinement is enabled, the converged geometry is submitted to MOPAC with a standard amide and peptide bond correction (see Figure 1F). An implicit aqueous solvent model (*ε *= 78.4) [30] is available to better match physiological conditions. The keywords used for the MOPAC run are MMOK, CHARGE = N (automatically determined for each molecule in the sanitization step, Figure 1B), XYZ, and OPT. For method comparison work, seven additional Hamiltonians are accessible: PM6, PM6‐D3H4X, PM6‐ORG, RM1, PM3, AM1, and MNDO. The heat of formation is stored as a molecular property. After refinement, a charge audit compares the MOPAC formal charge against the pre‐refinement value; a discrepancy triggers an automated rescue heuristic, and if that fails, the record is rejected rather than being passed on with a corrupted protonation state. On Windows, distribution MOPAC ships inside the executable and needs no separate installation.

Structures are exported as MOL2, SDF, or PDBQT (Figure 1G), either per compound or as a concatenated file ready to dock. Every run also produces a JSON audit report with per‐compound entries covering co‐former removal, ionization, stereocenter outcomes, embedding and optimization results, and semi‐empirical metadata, which aids troubleshooting of unexpected results.

To characterize the workflow under a realistic load, a library of 2020 compounds retrieved from the European Chemical Biology Database (ECBD) was processed at pH 7.4, together with a curated control set of nine drugs: two reference compounds (acetaminophen and ibuprofen), four ionization‐susceptible compounds (captopril, metformin, ciprofloxacin, and sulfamethoxazole), and three high‐complexity compounds (ritonavir, paclitaxel and cyclosporin). The control set returned 9/9 cleaned drug structures; ibuprofen was excluded by the strict stereocenter policy (Figure 2A) and expanded by the enumerative mode into two uniquely labeled stereoisomers (Figure 2B). Embedding succeeded on the first pass for every compound. On a single core of an Intel i7‐10750H processor with 16 GB of RAM, 1632 ligands from the ECBD set were prepared in 85 s when geometry refinement was restricted to the MMFF94 force field, a throughput of approximately 1263 molecules per minute; enabling PM7 refinement on the same set and hardware increased the wall‐clock time to 1.8 h, equivalent to 15.2 molecules per minute. Because the records are mutually independent, large libraries can be partitioned into disjointed shards processed by independent worker instances. On an eight‐core workstation, a separate 2000‐compound benchmark set processed in this data‐parallel mode reached an aggregate throughput rising from 342 molecules per minute on a single worker to 1722 on eight, a near‐linear 3.8‐fold speedup on four workers (94% parallel efficiency), and fivefold on eight. The absolute rate depends on molecular size and hardware, whereas the near‐linear scaling with worker count is the property relevant to large library preparation (Table 3).

**FIGURE 2:**
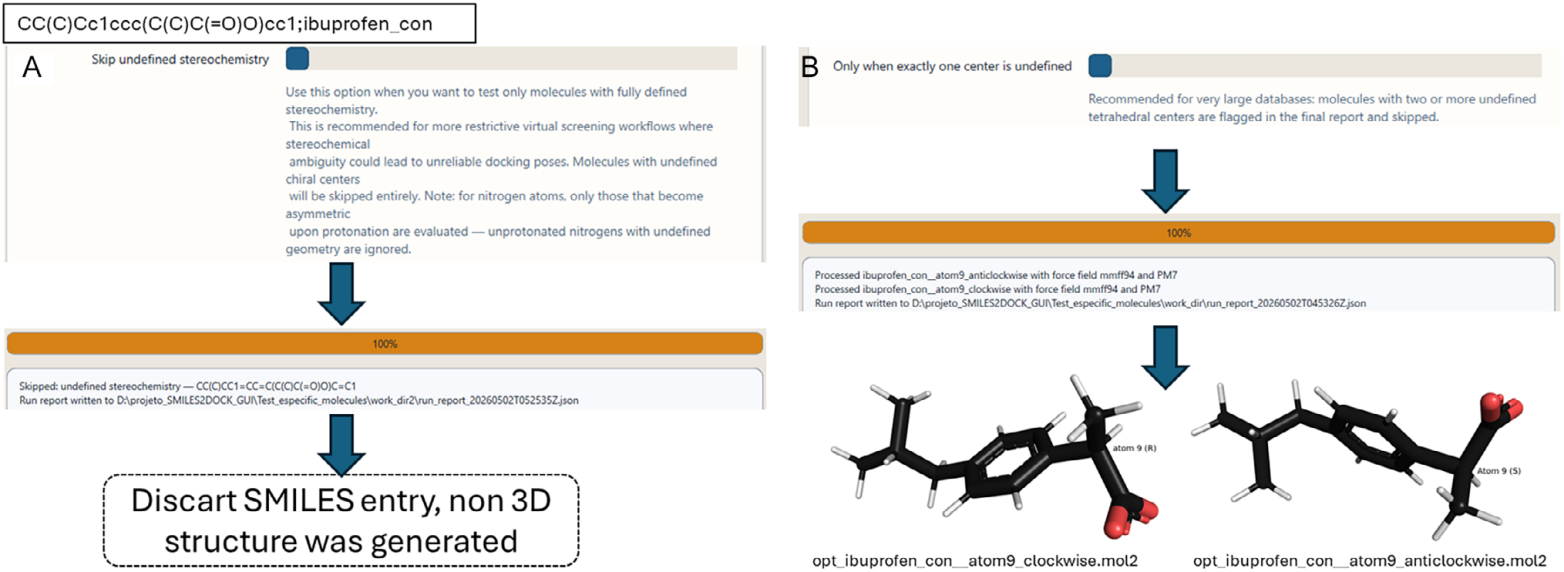
Strict (A) and flexible (B) functions for 3D structures generation with semi‐empirical refinement, Ibuprofen example. Atoms of hydrogen are shown in white, carbon in black and oxygen in red for three‐dimensional structures.

**TABLE 3:** Throughput of the force‐field preparation pipeline under data‐parallel sharding. A 2000‐compound benchmark set of drug‐like molecules was split into 200‐compound shards, each handled by an independent worker instance on an eight‐core workstation (Dimorphite‐DL ionization at pH 7.4, MMFF94 refinement, no semi‐empirical step). Throughput is the aggregate over all workers; efficiency is the speed‐up divided by the worker count. Values near unity indicate near‐linear scaling, and the marginal excess at two workers reflects run‐to‐run variance and CPU frequency scaling.

Workers	Molecules prepared	Wall‐clock, s	Throughput, mol min^−1^	Speed‐up	Efficiency
1	200	35.1	342	1.0×	100%
2	400	34.2	701	2.1×	103%
4	800	37.3	1287	3.8×	94%
8	1600	55.7	1722	5.0×	63%

Compared to a minimal RDKit‐plus‐Dimorphite‐DL pipeline, SMILES2Docking adds two controls targeting known failure modes. Stereocenter policies make chirality handling explicit and per‐compound auditable: standard RDKit embedding silently assigns an arbitrary configuration to an undefined stereocenter, whereas strict and enumerative modes filter or expand the compound. This matters because chirality errors propagate directly into binding‐affinity predictions [15]. The post‐refinement charge audit catches ionization changes that can occur during semi‐empirical optimization and either rescues the structure or rejects it since an incorrect charge reliably degrades docking scores [9].

Semi‐empirical refinement with PM7 is offered as an optional step and is disabled by default. It is not a prerequisite for docking, and it carries a substantial computational cost. On the ECBD set, enabling PM7 reduced the throughput from approximately 1263 to 15 molecules per minute on identical hardware. Its role is therefore framed as a user‐controlled refinement for small, curated series in which a quantum‐mechanically optimized geometry and internally consistent partial charges are desirable rather than as a stage in high‐throughput screening, for which the force field cascade alone is the recommended configuration. Although semi‐empirical refinement has been reported to improve pose prediction in specific cases, in particular for metal‐containing complexes and for systems with extended conjugation or delocalized charge, in which force‐field partial charges are less reliable [31, 32], such gains are system‐dependent, and no systematic improvement in docking scores is claimed here for PM7‐refined geometries. The choice is left to the user and is recorded, together with the Hamiltonian and solvation settings, in the audit report.

The tool is also aimed at researchers who do not have the time or programing background to assemble a custom preparation pipeline. ChEMBL, ZINC, and PubChem together hold very large numbers of compounds encoded as SMILES strings, and each step between a SMILES string and a docked pose can introduce errors when an unsuitable method is chosen; SMILES2Docking targets the careful, auditable preparation of curated and medium‐sized libraries rather than exhaustive ultra‐large screening, for which the docking stage rather than the preparation, is rate‐limiting. The three stereocenter modes reflect decisions that come up in practice: permissive mode for exploratory campaigns where the database contains many structures with undefined stereochemistry; strict mode when every output must be geometrically unambiguous; and enumerative mode when the scaffold is fixed, and all diastereomers need docking. Figure 2 displays two of the three cases: (i) strict restriction in undefined stereochemistry molecules, such as the SMILES representation of ibuprofen (Figure 2A) and (ii) enumerative mode, which generates all stereoisomers when one or more stereogenic centers are undefined (Figure 2B). There is also the flexible option when more than one stereogenic center is undefined, constructing up to 16 possibilities when applied.

The default backend returns a single dominant ionization state, predicted by the MolGpKa graph‐convolutional pKa model [24] and refined by iterative titration so that the coupling between neighboring ionizable centers is represented; rule‐based enumeration through Dimorphite‐DL [17] remains available when every plausible state is to be carried forward. An optional dominant‐tautomer step may be enabled before ionization. When RDKit is selected, its canonical tautomer is returned; when the externally installed sPhysNet‐Taut model [33] is provided, the tautomers are ranked by predicted aqueous free energy, the lowest‐energy tautomer is selected, and the protonation backend is then applied to it; sPhysNet‐Taut runs on Linux only. The MolGpKa model, being data‐driven, under‐predicts the pKa of small, unactivated aliphatic amines that are under‐represented in its training data. This out‐of‐distribution behavior is corrected by the per‐atom chemical prior described above, so that amines such as methylamine and tert‐butylamine are protonated as expected at physiological pH, whereas activated amines, whose low pKa is genuine, and amines coupled to a charged center are left to the model. A single machine‐readable report is written per run, with one entry per compound instead of one file per molecule, so the audit trail does not proliferate into many separate files. Preparation is computationally light, on the order of tens of milliseconds per molecule, and the records are mutually independent, so the workload is parallel: partitioning a library across independent worker instances scales the throughput near‐linearly with the available cores, and the same principle extends across the nodes of a compute cluster. For ultra‐large screens of 106–109 compounds, the rate‐limiting stage is the docking itself rather than the preparation, and platforms such as VirtualFlow add distributed job orchestration and an integrated docking engine rather than faster preparation. Codex (OpenAI) and Claude Code (Anthropic) were used to merge code, build the graphical interface, and revise the language.

## Author Contributions

**Daniel Andrés Grajales Ruiz** **:** conceptualization, data curation, methodology, writing – review and editing. **Bruna Flôres Negrisoli** **:** data curation, validation, writing – original draft preparation. **Isabel Cristina Conceição Periquito** **:** data curation, validation, writing – original draft preparation. **Nailton Monteiro do Nascimento‐Júnior** **:** supervision, data validation, writing – review and editing. **Adriano Marques Gonçalves** **:** conceptualization, data curation, methodology, writing – review and editing, supervision.

## Funding

This study was supported by Fundação Nacional de Desenvolvimento do Ensino Superior Particular, Conselho Nacional de Desenvolvimento Científico e Tecnológico (140246/2022‐3, 465.249/2014‐0), Fundação de Amparo à Pesquisa do Estado de São Paulo (2025/17931‐4, 2018/00187‐7), Fundação Carlos Chagas Filho de Amparo à Pesquisa do Estado do Rio de Janeiro (E26‐010.000090/2018).

## Conflicts of Interest

The authors declare no conflicts of interest.

## Data Availability

The data that support the findings of this study are openly available in Smiles2Docking at https://github.com/amgoncalvesusp/Smiles2Docking.
